# Psychological distress among Plains Indian mothers with children referred to screening for Fetal Alcohol Spectrum Disorders

**DOI:** 10.1186/1747-597X-5-22

**Published:** 2010-09-06

**Authors:** Tassy Parker, Marcello A Maviglia, Phyllis Trujillo Lewis, J Phillip Gossage, Philip A May

**Affiliations:** 1 Department of Family and Community Medicine, University of New Mexico School of Medicine, MSC09 5040, 1 University of New Mexico, Albuquerque, New Mexico, USA; 2 Center on Alcoholism, Substance Abuse, and Addictions, The University of New Mexico, 2650 Yale SE, Albuquerque, New Mexico, USA; 3 Department of Sociology, University of New Mexico, Albuquerque, New Mexico, USA

## Abstract

**Background:**

Psychological distress (PD) includes symptoms of depression and anxiety and is associated with considerable emotional suffering, social dysfunction and, often, with problematic alcohol use. The rate of current PD among American Indian women is approximately 2.5 times higher than that of U.S. women in general. Our study aims to fill the current knowledge gap about the prevalence and characteristics of PD and its association with self-reported current drinking problems among American Indian mothers whose children were referred to screening for fetal alcohol spectrum disorders (FASD).

**Methods:**

Secondary analysis of cross-sectional data was conducted from maternal interviews of referred American Indian mothers (n = 152) and a comparison group of mothers (n = 33) from the same Plains culture tribes who participated in an NIAAA-funded epidemiology study of FASD. Referred women were from one of six Plains Indian reservation communities and one urban area who bore children suspected of having an FASD. A 6-item PD scale (PD-6, Cronbach's alpha = .86) was constructed with a summed score range of 0-12 and a cut-point of 7 indicating serious PD. Multiple statistical tests were used to examine the characteristics of PD and its association with self-reported current drinking problems.

**Results:**

Referred and comparison mothers had an average age of 31.3 years but differed (respectively) on: education (<high school: 47.4%, 9.1%), PD-6 mean scores (3.57, 1.48), current prevalence of serious PD (19.1%, 0.0%), and a current drinking problem (31.6%, 12.1%). Among referred mothers, those with a current drinking problem had a significantly higher mean PD-6 score. Having PD, serious PD, and 2 specific scale items significantly increased the odds that a referred mother would have a current drinking problem.

**Conclusions:**

Psychological distress among referred mothers is significantly associated with having a self-reported drinking problem. FASD prevention requires multi-level prevention efforts that provide real opportunities for educational attainment and screening and monitoring of PD and alcohol use during the childbearing years. Mixed methods studies are needed to illuminate the social and cultural determinants at the base of the experience of PD and to identify the strengths and protective factors of unaffected peers who reside within the same communities.

## Background

Psychological distress (PD) is an unpleasant subjective state involving co-occurring mood and anxiety symptoms [1]. It is a non-specific condition that is associated with increased mortality [2], considerable emotional suffering and serious role impairments [3], increased somatization [4], and of specific interest to the current study, increased and heavier alcohol use [5]. Approximately 11% of the US adult population experience serious PD in the past year [6]; however, there are substantial differences in PD prevalence by race/ethnicity.

Among participants of the 2007 National Survey on Drug Use and Health [7], Asians had the lowest rate (6.4%) of serious PD in the past year and American Indians the highest (13.7%). Likewise, while less than one-half of the participants with serious PD received mental health services, there were major racial/ethnic differences with Whites being much more likely than minorities to receive care (51% vs 28%, respectively). Among adults aged 18 or older with past month serious PD in 2008, the rate of binge alcohol use (drinking five or more drinks on the same occasion on at least 1 day in the past 30 days) was 30.9 percent, which was higher than the 24.6 percent rate among adults who did not meet criteria for serious PD [7]. The rate of heavy alcohol use (drinking five or more drinks on the same occasion on each of 5 or more days in the past 30 days) among adults with serious PD in the past month was higher (12.1 percent) than the rate reported among adults without past month serious PD (7.3 percent).

The following summary points from the PD-associated literature frame the importance of PD research: 1) PD is associated with heavier alcohol consumption among women of childbearing age [5]; 2) women with PD who are also at risk for birth of a child with an FASD often continue to use alcohol despite knowing that they are pregnant and they have been advised against alcohol use [8]; 3) independent of alcohol use, maternal PD in the form of depression, anxiety, and stress is linked to elevated cortisol levels, which is in turn related to spontaneous abortion, preeclampsia, lower fetal weight and prematurity [9]; and 4) a significant risk factor for PD is unresolved psychosocial stress [1]. Regarding the latter point, some American Indian women have been found to be at increased risk for experiencing severe psychosocial stress due to such prevalent conditions as the high rates of female-headed households, large families, lower high school graduation rates, the greater likelihood of living below the poverty level, fair or poor health status [10,11], and an elevated lifetime risk of traumatic interpersonal violence [12].

It follows then that many American Indian women would be at increased risk for serious PD. In fact, during 2000-2004, American Indian women were reported to have a past 30 day PD prevalence of 9.7%, a rate at least two times that of Black women (4.0%), White women (3.4%), and Asian women (2.4%) [13]. They also have the highest mean number of days per month of feeling sad, "blue", or depressed [14]. In addition, American Indian women have more than twice the past 30-day prevalence of serious PD as American Indian men (3.9%) [13].

Also relevant is a recent finding that the prevalence of past month binge drinking among American Indian women is higher than that of non-American Indian women in the U.S. (9.6%, 7.5%, respectively). However, neither group of women meet the Healthy People 2010 goal of reducing binge drinking to 6% [11].

This PD study took place within a larger NIAAA-funded epidemiology and community-wide prevention study of fetal alcohol spectrum disorders (FASD). In this program, diagnosis and comprehensive prevention of FASD were carried out in a time-sequenced manner to reduce the prevalence of FASD, first in four reservation communities of the Northern Plains and then in two additional reservations sites and one urban catchment area. FASD diagnostic clinics were held regularly in order to identify cases and families in need of intervention and prevention services. Rates of FAS have been reported in the literature to be between 8 and 9 per 1,000 children on Plains culture reservations which is approximately 2 to 4 times the estimates for the U.S. general population [15].

In addition, in the larger investigation in which this PD study took place, depression and anxiety seemed common. Although drinking in general among American Indian women of the six reservation study communities was not significantly more prevalent than in the general population of the U.S., binge drinking was very high among those who chose to drink [16]. Furthermore, our clinical investigations, and those of others [8], clearly indicated the presence of various symptoms of depression and PD among mothers whose children were diagnosed with an FASD.

The examination of maternal risk factors for FASD and PD-associated risk factors, and our knowledge of the prevalence of these risk factors in the landscapes of American Indian women of the Plains challenged us to more fully describe any association between PD and problem drinking. Especially important is to explore this association among Plains Indian women in their childbearing years and particularly among those women who may already have given birth to a child with FASD. Therefore, while a small comparison group of mothers is initially introduced as a means of providing additional context, the main focus of the study is on the referred mothers. Mothers of children with FAS are at greater risk for having more children with FAS [17]. If mothers continue to drink with successive pregnancies, children born later often have more profound effects of FAS [18]. In the only study to date that looked at American Indian siblings born before and after a child with FAS, it was found that the FAS characteristics of the siblings born before could have predicted the subsequent alcohol affected sibling and that the siblings born after also had significant central nervous system impairments [19]. A study involving children of other cultures found that over 40% of the children diagnosed with FASD had one or more sibling who also carried an FAS diagnosis [20].

In explaining the treatment of PD for use in FASD prevention, it is our hope to increase awareness about the need for treatment to decrease the psychosocial burdens of PD on American Indian mothers in general and to provide empirical evidence for the development of effective prevention and policy that might support the health of American Indian mothers and their children. Therefore, this investigation explores the following questions: 1) What is the current prevalence of PD and drinking problem among referred mothers?, 2) Are there significant differences in prevalence of current drinking problem and PD mean scale score between the referred and comparison groups of mothers?, 3) Do PD scale scores significantly differ by drinking status among referred mothers?, and 4) What are the associations between various characteristics of PD such as PD in general, serious PD, and individual PD scale items, and the potential of having a current drinking problem among referred mothers?

## Methods

All research and interventions in this study were carried out under the authority of resolutions from the participating tribal councils and with ongoing approval of a variety of human subjects review boards. Protocols and consent forms used were approved by The University of New Mexico (Medical School HRRC 96-209 and 00-422, and the main campus IRB 9625), three Indian Health Service (IHS) IRBs, and the NIH Office of Protection from Research Risks (OPRR). All women gave active consent before providing information or otherwise participating in the main study and in this sub-study.

### Sample Characteristics

Women who are the focus of this paper were recruited from multiple community sources (clinics, schools, and social service agencies) and were originally identified via their children's enrollment in a NIAAA-funded comprehensive fetal alcohol syndrome (FAS) research and prevention project (referred to as Fetal Alcohol Syndrome Epidemiological Research - FASER). The FASER project took place in six Northern Plains Indian communities and one urban area in the Plains. To test for possible differences between referred mothers residing on the participating reservations and those mothers residing in the urban location (12% of the referred mothers' sample), we compared the means or percentages on each key variable with and without the urban mothers subsample and found no statistically significant differences. Therefore, we included the urban group in our analyses. All female participants in the study self-identified as American Indian of a Plains or Plateau cultural heritage.

Children were referred to the study if they had a previous diagnosis of FAS, another FASD, or fetal alcohol effect (FAE), if a school, medical, or social service official suspected that a particular child had been exposed to alcohol during the mother's pregnancy (e.g., the index pregnancy), or if a child was having difficulty in school, either academically or behaviorally. The referred children were seen in special developmental clinics and provided dysmorphology examinations by physicians specifically trained in FASD diagnostics and human genetics. A short while later, the referred children were administered a battery of educational tests, and the mothers of the referred children were interviewed by FASER staff.

Children, matched on age and gender with 33 participating children in their respective communities who were not suspected of having an FASD, were recruited via flyers and word of mouth by project coordinators from local health clinics and other locales within the community for comparison. The recruitment of the comparisons emphasized that the children were to be considered as "normally developing" children within these communities. These comparison children were given the same educational tests, and the mothers were also interviewed concerning risk or protective factors during the index pregnancy.

One hundred eighty-five women were included in the particular sub-study reported on here: 152 women whose children were referred because their children were suspected of having an FASD (the children of 23 or 15.1% of these referred mothers were found to have an FASD),); in this comparison there are 33 comparison women whose children were recruited specifically because their children were believed to have no identified physical or learning/behavioral problems, and the diagnostic process confirmed this to be the case. Furthermore, the maternal interviews administered to the mothers of the comparison children confirmed that they engaged in little drinking in the child's prenatal period or in the present.

### Research and sampling procedure

Data for this study are derived from the FASER comprehensive prevention trial. Beyond the baseline and ongoing epidemiological research, the ultimate aim of FASER was to demonstrate that fetal alcohol syndrome can be prevented by the application of the FAS prevention model put forth by the U.S. Institute of Medicine (IOM) in 1996 [18]. It utilizes a comprehensive, community-wide approach by applying techniques of universal, selective, and indicated prevention in a control study of six Plains communities. Details about the FASER study methods are available in other literature [16,21,22].

### Measures

Within the main study, maternal risk factor data were collected via an extensive questionnaire containing over 200 items pertaining to socio-demographics, general health, pregnancy history, nutrition, tobacco, alcohol, and drug use. Some of the demographic and alcohol data were used for this sub-study, including age (a continuous variable), and education (a categorical variable ranging from less than graduation from high school or receiving a GED (general educational development diploma)), to some college or a college degree. Alcohol use (by quantity, frequency, and timing) was determined by self report after the administration of an extensive set of alcohol consumption questions for the past week, past month and year, and also during the three months before and during each trimester of the index pregnancy. After reflecting on those questions and her own drinking behavior, the respondent was presented with the question "Do you currently have a drinking problem?". Response options included "yes" or "no". Thus, it was each woman's self report, in response to this single measure that she did or did not have a current drinking problem, that was used in the analysis of the data.

In the scientific literature, psychological distress (PD) is measured in a number of ways. Some studies use a combination of measures for depression, anxiety, and daily hassles [9]. Others define PD by its attributes such as a perceived inability to cope effectively, change in emotional status, discomfort, communication of discomfort, and harm [23]. In several US national health studies PD is measured by symptoms of depression and anxiety that are known to be associated with dysfunction in daily life, particularly in social functioning, and is used as an indicator of health service needs. In the parent FASER study, psychological distress was assessed with the use of the Self-Report Symptoms Checklist (SSCL-51) developed by Uhlenhuth, et al. [24] used to measure common psychological symptoms in clinical populations. It is a slightly modified version of the Hopkins Symptom Checklist (HSCL) by Derogatis, et al. [25]. The total score represents a self-report of how much distress the person has experienced in the past month. Separate scale scores indicated the ways and the extent to which the person is distressed: (i.e., not at all, a little trouble, or a lot of trouble).

In the study reported here, PD is measured with a 6-item scale that was constructed from the SSCL-51 included in the FASER maternal survey and that we will now refer to as the PD-6. Items from the SSCL-51 were selected for the PD-6 based on their similarity to items used in the K6 [26], a nationally recognized and reliable measure of PD. The K6 is used in large-scale health surveys in the US such as the National Health Interview Survey and the National Survey on Drug Use and Health to estimate serious mental illness in the U.S. general population. A description of the PD-6 as well as a comparison of PD-6 items with those items included in the K6 is presented in Table 1. As noted the PD-6 response scale has a range of 0-2 compared to the 0-4 range of the K6. In addition, while both scales use one point above the middle value of the summed score range as the cut point score for serious PD, that value was determined in substantially different ways. The K6 cut point score for serious PD was derived through advanced psychometric testing including the examination of receiver-operating characteristic (ROC) curves to determine the sensitivity and specificity of distinguishing normal from distressed in multiple and large community samples [26]. In the current study, secondary analysis and a fixed sample limit the testing of the chosen cut point. Nevertheless, the cut point and operationalization of serious PD for the purpose of this study demonstrated distinguishing characteristics in relation to other key variables. Reliability of the PD-6 scale is good (Cronbach's alpha = 0.86).

**Table 1:** Stem statement and items for PD-6* Scale and K6** Scale

PD-6 Scale:	K6 Scale:
"How much trouble have you had with each of these things in the past month?"	"During the past 30 days, how often did you feel ..."

1) feeling blue or down in the dumps or depressed	1) so sad that nothing could cheer you up?

2) feeling nervous, fidgety, tense	2) nervous?

3) being so restless I can't sit still	3) restless or fidgety?

4) feeling hopeless about the future	4) hopeless?

5) having trouble getting myself going	5) that everything was an effort?

6) feeling worthless	6) worthless?

*3-point response scale (0 = not at all, 1 = a little trouble, 2 = a lot of trouble)

** Kessler et al. (2002), K6, 5-point response scale (ranging from 0 = none of the time to 4 = all of the time)

### Analytic approach

Descriptive statistics were used to identify sample characteristics and PD mean scale scores. Prevalence of serious PD was determined as being the proportion (%) of women with a FASER PD-6 summed score ≥7. Mean differences for the key variables by referral and comparison mothers were calculated with the chi-square test statistic for the categorical measure and, because of the substantial difference in group sample sizes, the Mann-Whitney U test statistic for continuous measures. A two-tailed, independent samples *t*-test was conducted to assess the differences in mean PD-6 scores by current drinking problem (yes, no) among referred mothers. Although the test group sizes were unequal, Levene's test for equality of variance was non-significant, thus signifying an appropriate use of the *t*-test.

Logistic regression was used to calculate bivariate and multivariate odds ratios and confidence intervals were calculated to provide an estimate of the risk for self-reported current drinking problem in association with PD and serious PD. We also conducted the same analyses for each of the PD-6 individual items to better understand the specific PD symptoms that are most troublesome. In preparation of the logistic regression used to produce the odds ratios for the individual PD-6 items we first examined the cell sizes of the response categories and found relatively small cell sizes (ranging from 4.6%-19.1%) for the response option "a lot of trouble". Questioning about mental health symptoms can provoke a perception of social stigma, particularly in the present study whereby the survey was conducted face-to-face in contrast to a more discreet method such as anonymous, self-administration. Therefore, it is possible that social desirability (responding in a way to avoid criticism or judgement) and or social approval (responding in a way that seeks praise) response set biases may have been present in the mothers' answers to the PD questions, as has been reported for other populations and conditions [27-30]. Thus, it may be the case that the referred mothers who reported having even "a little trouble" may in fact be experiencing substantially more trouble. For the purpose of the current exploratory examination of the individual PD items, binary response categories were constructed for each item as follows: 0 = not at all and 1 = a little trouble/a lot of trouble. Thus, our analysis of the individual items considered the odds ratios for self-reported drinking problem on the basis of the women reporting not having trouble with a PD symptom at all or having a little to a lot of trouble with a PD symptom. All data analyses were conducted using SPSS for Windows, Version 16.0 [31].

## Results

Descriptive statistics for the key variables are presented in Table 2. While both referred and comparison mothers have an almost identical mean age of approximately 31 years they differed significantly on education. Almost one-half of referred mothers had less than a high school education. In response to research question 1, "What is the current prevalence of serious PD and drinking problem among referred mothers?", Table 2 shows that the current prevalence rates of self-identified drinking problem and serious PD among referred mothers are 31.6% and 19.1%, respectively. In addition, in response to research question 2, "Are there significant differences in prevalence of current drinking problem and PD mean scale score between the referred and comparison groups of mothers?", Table 2 indicates that a significantly greater percentage of referred mothers have a current drinking problem and they also have a significantly higher PD-6 mean scale score. Using a PD-6 summed score cut-point of 7 to indicate serious PD, approximately 19% of referred mothers and none of the comparison mothers have serious PD.

**Table 2:** Descriptive and nonparametric test statistics for key variables by referred and comparison mothers

Variables	Referred Mothers (N = 152)	Comparison Mothers (N = 33)	**Test Statistic**^**a**^
Age (s.d., years)	31.3 (7.38)	31.3 (6.64)	U = 2438.5, z = .250

Education (%):			χ^2^(2) = 29.32***

<high school	47.4	9.1	

high school/GED	29.6	21.2	

some college/college degree	23.0	69.6	

Self-reported current drinking problem (%)	31.6	12.1	χ^2^(1) = 5.08*

PD-6:			

mean scale score (s.d.)	3.57 (3.10)	1.48 (1.39)	U = 1492.5, z = 3.68***

% at cut point ≥7 (scale range: 0-12)	19.1	0	

^a ^Mann-Whitney U, Pearson's chi-square

*p < .05, **p < .01, ***p < .001

The results presented in Table 3 answer research question 3, "Do PD scale scores significantly differ by drinking status among referred mothers?". Referred mothers who reported having a current drinking problem had a significantly higher PD-6 mean scale score than referred mothers without a drinking problem.

**Table 3:** Referred mothers' PD-6 mean scale scores by current drinking problem (N = 152)

Current Drinking Problem	PD-6 Mean Scale Score (s.d.)*
Yes (n = 48)	4.35 (3.31)

No (n = 104)	3.21 (2.94)

*2-tailed *t*-test, *T(150) *= 2.14, p ≤ .05

With a continued focus on referred mothers, the odds ratios reported in Table 4 respond to the final research question, "What are the associations between various characteristics of PD such as PD in general, serious PD, and individual PD scale items, and the potential of having a current drinking problem among referred mothers?". Bivariate analyses indicate that PD significantly increases the odds of a self-reported current drinking problem while serious PD (a PD-6 score of at least 7) multiplied the odds of a self-reported current drinking problem by almost two and a half times. In addition, both depression (hopeless about the future) and anxiety symptoms (nervous, fidgety, tense) more than doubled the odds of self-reported drinking. When controlling for the available demographics, age and education, those same findings remain significant with comparable multivariate and bivariate odds ratios.

**Table 4:** Referred mothers' bivariate and multivariate odds ratios for current drinking problem by PD-6

Variable	**Bivariate Odds Ratio**^**a**^ (95% CI)	**Multivariate Odds Ratio**^**a,b **^ (95% CI)
PD-6 summed score	1.12* (1.01 - 1.25)	1.15* (1.02 - 1.28)

PD-6 summed score ≥7	2.44* (1.07 - 5.60)	2.60* (1.19 - 6.03)

Individual PD-6 items^c^		

1) feeling blue or down in the dumps or depressed	1.55 (0.73 - 3.29)	1.60 (0.75 - 3.41)

2) feeling nervous, fidgety, tense	2.16* (1.06 - 4.41)	2.32* (1.12 - 4.80)

3) being so restless I can't sit still	1.48 (0.72 - 3.05)	1.59 (0.73 - 3.19)

4) feeling hopeless about the future	2.19* (1.08 - 4.41)	2.39* (1.16 - 4.91)

5) having trouble getting myself going	1.85 (0.92 - 3.71)	1.90 (0.93 - 3.87)

6) feeling worthless	1.47 (0.72 - 3.01)	1.58 (0.76 - 3.26)

^a ^logistic regression

^b ^controlling for the effects of age and education

^c ^binary response scale used: 0 = not at all, 1 = a little trouble/a lot of trouble

* p ≤ .05

## Discussion

Despite compelling evidence about the elevated prevalence of serious PD among American Indian women compared to women of other racial/ethnic groups in the U.S., and the numerous validations of PD-related social- and health-related consequences including increased binge and heavy alcohol use, this study is a first effort to examine PD among Plains Indian women in conjunction with FASD epidemiology and prevention. The PD-6 scale, created solely for the current investigation and thus with some limitation, was a reliable measure of PD among the American Indian women in our study and provided us with the means for examining associations between PD and maternal drinking.

We identified mean scores of past 30-day PD, prevalence of serious PD, and prevalence of self-reported current drinking problem among Plains Indian mothers with children referred for FASD screening within a context that included a small comparison group of Plains Indian mothers of unexposed and unaffected children. The women, who lived in the same communities, shared a common tribal culture, and had a common mean age, were markedly different in three important ways as shown in Table 2. First, referred mothers were five times more likely than the comparison mothers to have less than a high school education. There is ample evidence that social disadvantage is highly relevant to psychological distress and problem drinking [32]. Second, PD mean scores were significantly different between the two groups with serious PD affecting almost one-fifth of the referred mothers but none of the comparison mothers. Third, referred mothers were almost three times as likely as the comparison mothers to have a self-reported current drinking problem.

Within the group of referred mothers, we found a significantly higher PD-6 mean score for mothers with a self-reported current drinking problem (Table 3). Not only is serious PD prevalent among the American Indian mothers with children referred for FASD screening, the results reported in Table 4 indicate that having serious PD significantly increased the potential that the mothers already at risk have a current drinking problem, even when controlling for the effects of age and education on having a drinking problem.

From a prevention perspective and specific to the prenatal care setting where routine depression screening is gaining ground nationally, the PD associated with alcohol use may be missed if symptoms of anxiety are not also assessed in the presence of depression. As was discovered (Table 4), women who were feeling hopeless about the future (depression) and were troubled by being nervous, fidgety, or tense (anxiety) had significantly increased odds of having a self-reported current drinking problem. Therefore, use of a PD screening tool may cast a wider prevention net for identifying a potential threat of alcohol use than would a depression screener alone.

While we emphasize the role of PD as an important consideration for the prevention of maternal drinking, we also point out that the readiness of accessible, effective treatment for depression, anxiety, and stress does not correspond to the women's well-documented need. A disproportionate mental health burden for American Indian women with PD includes a substantially higher self-reported unmet need for mental health treatment as compared to women of all other major racial/ethnic groups [33]. When mental health treatment is received, it is not likely to be evidence-based [34,35] or culturally sensitive [36]. Furthermore, American Indian women have not been included in any of the numerous national depression treatment studies, nor has the vast body of knowledge that cultural variables affect diagnosis, help-seeking, course, treatment, and patient satisfaction with treatment [37-40] yet been translated into beneficial treatment for American Indian women who suffer psychological distress [41,42].

Effective treatment for PD and co-occurring conditions such as alcohol abuse is even more elusive. However, the lack of such treatment is not specific to American Indian communities alone. In the general population, co-occurring alcohol use and PD are under diagnosed and inadequately treated [43,44]. Among low-income ethnic minorities, the unmet need for adequate treatment is even greater than among the general population [45]. However, American Indian communities can promote the development of culturally-tailored and effective treatments for women with co-occurring risk factors such as PD and problematic alcohol use through research partnerships that include the affected women in respectful consultation and meaningful participation.

### Limitations and Future Research

Our study is limited by several factors. The first consideration is the cross-sectional design that prevents us from establishing the causal relationship between PD and self-reported current drinking problem among the women. Our construction of the issue contends that PD increases the potential of having a current drinking problem. However, it is also likely that having a drinking problem leads to or exacerbates PD as was the finding in a recent longitudinal study of the relationship between cocaine and methamphetamine use and PD [46]. A second limitation was the relatively small size of the comparison group of mothers that hampered further statistical analysis and comparative study of intergroup differences. However, since our focus is on preventing maternal drinking, it is the at risk or referred mothers with whom we were most concerned.

An important outcome of our comparison of the two groups of mothers is the direction that it suggests for future studies. Of particular benefit for prevention efforts would be an investigation that identifies the assets and protective factors of Plains Indian women who reside in the same communities, but for whom serious PD and alcohol use are not co-occurring issues. Such factors could include further examination of this study's finding that the comparison group of mothers who were three times more likely than the referred mothers to have at least some college did not meet the established cut point criteria for serious PD. In addition, qualitative studies with Plains Indians and other American Indian mothers who may be at risk for maternal drinking can contribute to our understanding of the relationship between PD and maternal drinking. For instance, maternal narratives may reveal internal and external resources for coping with PD that are suggestive of intrinsic culture- and community-based strategies for FASD prevention that are contextualized to the women's real life experiences. From the parent FASER study, some earlier data on psychological distress, socioeconomic status (SES), and maternal risk factor variables have been identified [47]. With a larger sample, multiple correlation studies and models of the contribution of PD, SES, and other contextual variables of risk that lead to maternal drinking and high risk for children with an FASD are needed.

## Conclusion

A significant association exists between PD and a self-reported current drinking problem among Plains Indian mothers who may already be at risk for having a child with FASD. The social disadvantage of these women is confirmed by their low educational attainment. For tribal and other communities that are seeking solutions to maternal drinking, including clearly defined goals and programming for female educational achievement and occupational opportunity may increase the benefits of their FASD prevention efforts and policy. Routine, concurrent screening of women of childbearing age for psychological distress and alcohol use can provide important information to the health care provider about the potential need for active monitoring before and during pregnancy. An important caveat to our study is that while a quantitative measure of PD seems to capture the signs of the emotional experience of American Indian women, a parallel process of qualitative inquiry is also needed in order to illuminate the social and cultural determinants at the base of the experience of PD itself.

## Competing interests

The authors declare that they have no competing interests.

## Authors' contributions

TP and MAM constructed the PD measures from the FASER data base and conducted the secondary analyses. TP took primary responsibility for writing the first drafts of the paper. MAM helped write the discussion and conclusion sections of the paper. All data were originally collected in the FASER project by a large team and network of NIAAA-funded individuals listed in the acknowledgements. PTL conducted the majority of the maternal interviews in the seven communities. JG was the primary manager and monitor of all data collection, data entry, data set construction, and also compliance with IRB requirements. PAM was the Principal Investigator on each of the three NIAAA grants and the supplement that funded TP's work on this particular part of the study; he oversees all aspects of the FASER research from conceptualization to methodology to the relationships with the study communities, tribal councils, and NIAAA. All authors read, and contributed to the writing, and approved the final draft of the manuscript.
